# Spatial-Temporal Epidemiology of Tuberculosis in Mainland China: An Analysis Based on Bayesian Theory

**DOI:** 10.3390/ijerph13050469

**Published:** 2016-05-05

**Authors:** Kai Cao, Kun Yang, Chao Wang, Jin Guo, Lixin Tao, Qingrong Liu, Mahara Gehendra, Yingjie Zhang, Xiuhua Guo

**Affiliations:** 1 School of Public Health, Capital Medical University, No. 10 Xitoutiao, You’anmen Wai, Fengtai District, Beijing 100069, China; anzhen602@163.com (K.C.); yangkun_1123@163.com (K.Y.); 13810147054@139.com (C.W.); guojin5827501@163.com (J.G.); taolixin.2008@163.com (L.T.); liuqqa225@163.com (Q.L.); gbmahara@163.com (M.G.); 2 Beijing Municipal Key Laboratory of Clinical Epidemiology, Beijing 100069, China; 3 Beijing Ophthalmology & Visual Science Key Lab., Beijing Institute of Ophthalmology, Beijing Tongren Eye Center, Beijing Tongren Hospital, Capital Medical University, Beijing 100730, China; 4 Department of Statistics and Information, Beijing Centers for Disease Control and Prevention, No 16, Hepingli Middle Street, Dongcheng District, Beijing 100013, China; 5 Chinese Center for Disease Control and Prevention, Beijing 102206, China; cksgs2016@163.com

**Keywords:** tuberculosis, Bayesian theory, spatial-temporal interaction, ecological factors

## Abstract

*Objective*: To explore the spatial-temporal interaction effect within a Bayesian framework and to probe the ecological influential factors for tuberculosis. *Methods*: Six different statistical models containing parameters of time, space, spatial-temporal interaction and their combination were constructed based on a Bayesian framework. The optimum model was selected according to the deviance information criterion (DIC) value. Coefficients of climate variables were then estimated using the best fitting model. *Results*: The model containing spatial-temporal interaction parameter was the best fitting one, with the smallest DIC value (−4,508,660). Ecological analysis results showed the relative risks (RRs) of average temperature, rainfall, wind speed, humidity, and air pressure were 1.00324 (95% CI, 1.00150–1.00550), 1.01010 (95% CI, 1.01007–1.01013), 0.83518 (95% CI, 0.93732–0.96138), 0.97496 (95% CI, 0.97181–1.01386), and 1.01007 (95% CI, 1.01003–1.01011), respectively. *Conclusions*: The spatial-temporal interaction was statistically meaningful and the prevalence of tuberculosis was influenced by the time and space interaction effect. Average temperature, rainfall, wind speed, and air pressure influenced tuberculosis. Average humidity had no influence on tuberculosis.

## 1. Introduction

Tuberculosis (TB) remains a major public health burden in a number of developing countries. China alone accounted for nearly 1 million or an estimated 12% of the total TB cases reported worldwide in 2010 [1]. For each case of TB, the average diagnosis and treatment cost is nearly 2% of urban residents’ average annual income in mainland China [2]. TB has been ranked among the top five notifiable infectious diseases for decades [3] and more effort is needed to control and prevent it. Various factors influence the prevalence of TB, including demographic factors [2,4,5,6,7,8], medical resources [9], economic reasons [6,10,11], gene polymorphism [6,7,12,13,14], and behavior [9,15,16,17,18]. Yet the effect of the environment, especially climatic variables that can strongly affect the living environment of bacteria, are often overlooked [19,20]. Li *et al.* [21] explored ecological factors associated with spatial heterogeneity of TB in China, however, the data used were relatively old (2001–2010). In recent years, Bayesian models have been widely applied to the analysis of data containing both time and space information. Lamichhane *et al*. [22] fitted a space-time Poisson regression model within a Bayesian framework to deal with a complex spatial-temporal correlation structure in a store locations study in the USA. Naithani *et al*. [23] quantified leaf area index and volumetric soil water content spatial-temporal patterns using a hierarchical Bayesian model. In China, Bauer *et al*. [24] developed a model to assess the risk of hand, foot, and mouth disease by marginal spatial, temporal, and space-time interaction dimensions. In this study we applied a Bayesian model to explore whether time and space has an interaction in the prevalence of TB in China, and to assess whether climate variables are associated with the prevalence of TB.

## 2. Methods

### 2.1. Data Sources

Annually reported TB cases (2009 to 2013) in 31 mainland China provinces were obtained from the National Population and Health Science Data Sharing Platform [25]. Climate factor data, including average temperature, humidity, air pressure, rain fall and wind speed, was collected from the China Meteorological Data Sharing Service System [26].

### 2.2. Statistical Methodology: Bayesian Methods and Negative Binomial Distribution

Bayesian methodology is a widely used mathematical technique for data combining time and space information, while in traditional statistical methods no parameter was set to reflect spatial-temporal interaction effect. Bayesian theory uses sample information and prior distribution information to estimate posterior distribution parameters. This process can be done using Markov chain Monte Carlo (MCMC) methods in software such as WinBUGS. The negative binomial model is commonly used in infectious diseases research [27,28,29,30,31,32,33]; the function is shown in Equation (1):

(1) $$f\left(Y_{\mathrm{ij}}=y_{ij}|r,\mu_{\mathrm{ij}}\right)=\frac{\left(y_{ij}+r-1\right)!}{y_{ij}!\left(r-1\right)!}{\left(\frac{r}{r+\mu_{ij}}\right)}^{r}\times \;{\left(\frac{r}{r+\mu_{\mathrm{ij}}}\right)}^{y_{ij}},r>0$$

The number of cases, *y_ij_*, is assumed to follow a negative binomial distribution, with mean, dispersion parameter (*r*) and probability density. Variance of the counts, *var*(*y_ij_*) is assumed to be equal to:

(2) $$var(y_{ij})=\mu_{ij}\;+k\times \mu_{ij}$$

where *k* = 1/*r*, known as the aggregation parameter. The Poisson distribution arises as *k*→0 and thus *var* (*y_ij_*) =$\;\mu_{ij}$. The function that we applied consisted of two parts: the effect of population and the relative risk for each region. *e_ij_* stands for the expectation number of *i* province in *j* year and *θ_ij_* stands for the relative risk of *i* province in *j* year. The model was built as:

(3) $$\;\;y_{ij}\sim NB\left(\mu_{ij},r\right),\mu_{ij}=e_{ij}\theta_{ij}$$

Specifically, six models were constructed by considering time effect, spatial effect, and the interaction between time and space (Table 1).

The full model containing both spatial-temporal interaction and ecological parameters was built as:

*θ_ij_* = *exp*(α_0_ + *u_i_* + *v_i_* + *g_i_* + *psi_i,j_* + *α_1_ × time_1j_* + *beta1×precipitation_i,j_ + beta2 × airpressure_i,j_ + beta3 × windspeed_i,j_ + beta4 × temperature_i,j_ + beta5 × humidity_i,j_)*

### 2.3. Model Selection

The model with the smallest DIC value [34,35] was recommended according to the deviance information criterion. DIC value was recorded when the model iteration process reached stability, which could be evaluated by three kinds of plots: density, iteration history, and autocorrelation plots. To quit the iteration process, the density plot was expected to be an approximate normal distribution, the iteration history plot should fluctuate around a straight line and the autocorrelation plot should show an autocorrelation function quickly reaching zero.

### 2.4. Statistical Analysis Software

Statistical analysis was performed with ArcGIS (version 10.3, ESRI, Inc., Redlands, CA, USA), and WinBUGS (version 1.4.3, MRC Biostatistics Unit, Cambridge Biomedical Campus, Cambridge Institute of Public Health, Forvie Site, Robinson Way, Cambridge CB2 0SR, UK). ArcGIS software was used for descriptive analysis and WinBUGS was applied for Bayesian model iteration.

## 3. Results

### 3.1. Descriptive Analysis

**Figure 1:**
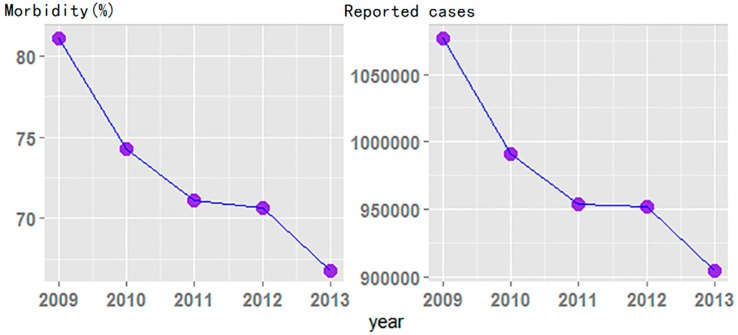
Trends in prevalence of tuberculosis in mainland China, 2009–2013.

**Figure 2:**
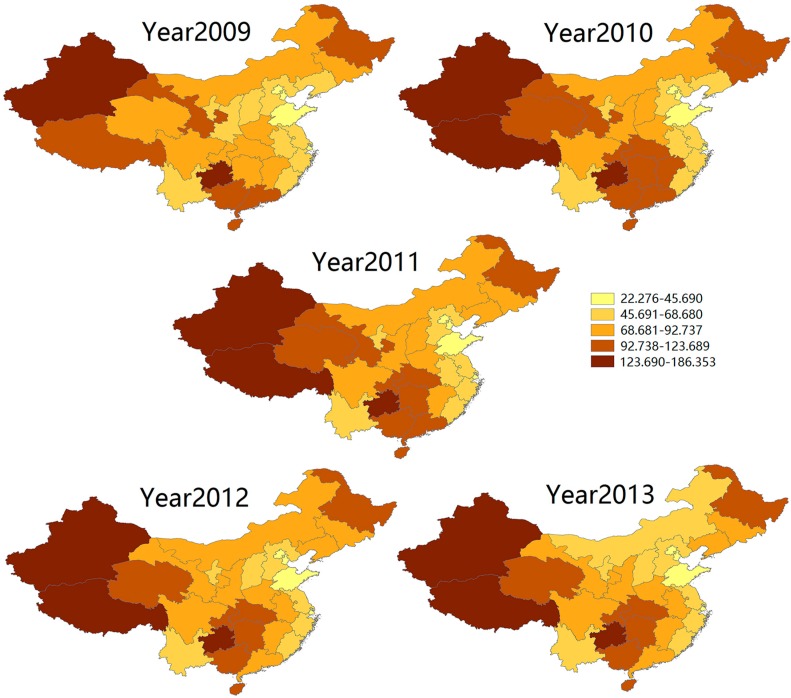
Spatial distribution of morbidity associated with tuberculosis in mainland China, 2009–2013.

As shown in Figure 1, reported cases of TB in China declined year by year, yet there were still 994,434 new cases in 2013. A typical spatial distribution tendency was also found among all the provinces (Figure 2). Specifically, Xinjiang Uygur, Xizang Province and Guizhou Province were the three areas of highest TB prevalence in China, with an average morbidity of 145.55 per 100,000 population. Coastal provinces in East China, particularly Shandong Province, had a low prevalence of TB, with an average morbidity of 40.37 per 100,000 population. For provinces in Central China, such as Henan, Shanxi, and Hebei, the morbidity of TB was between that of the western and eastern areas.

### 3.2. Exploration of Climate Factors

Comparison results among different models showed that the DIC values of the three uncorrelated effect (UH) models were −316, −530,089 and 1170, respectively. DIC values of the three correlated effect (CH) models were −562,338, −1800 and −4,508,660, respectively. The latter three were relatively small, indicating that model fit of CH models was better than that of UH models. Among the three CH models, the model taking the spatial-temporal interaction effect into consideration was the best fitting one, with a smallest DIC of −4,508,660.

Estimated coefficients (mean value) of temperature, rainfall, wind speed and air pressure were 0.00324, 0.01005, −0.18010 and 0.01002, respectively (Table 2). Correspondingly, the estimated *θ* (relative risk, *RR*) for these climate variables were 1.00324 (95% CI, 1.00150–1.00550), 1.01010 (95% CI, 1.01007–1.01013), 0.83518 (95% CI, 0.93732–0.96138) and 1.01007 (1.01003–1.01011). For these four variables, 95% confidence interval did not include 1, meaning that they were statistically significant. Temperature, rainfall, and air pressure had a positive influence on the prevalence of TB, while wind speed had the opposite effect.

The average humidity result showed an estimation value was −0.02535, and the corresponding *RR* was 0.97496 (95% CI, 0.97181–1.01386). With 95% confidence interval covering 1, the coefficient of humidity was not statistically significant.

## 4. Discussion

TB prevalence in mainland China has been decreasing continuously from 2009 to 2013, and the number of reported new cases declined from 1,076,938 to 904,434. The prevalence of TB in different areas of China varied considerably and the spatial distribution showed a typical hierarchical structure: high-level morbidity in the western regions, middle-level morbidity in central areas and low-level morbidity in the eastern part. This finding is similar to the epidemic pattern reported by the China Tuberculosis Control Collaboration in a national survey [36]. Still more attention should be paid to the western areas and appropriate medical resources should be allocated to this region, especially Xinjiang Uygur, the Xizang Autonomous Region and Guizhou Province.

Before ecological influential factors of TB were explored, a comparison among different models was performed. A Bayesian model containing the spatial-temporal interaction effect parameter fitted the best, meaning that an interaction effect existed between time and space. The prevalence of TB in mainland China was not only time-dependent (such as seasonal trend and long period trend) or merely spatially clustered; time and space reciprocally affect each other during the dynamic process of infectious disease transmission, which has also been observed in previous studies [37,38,39].

Estimations from the Bayesian model showed that temperature could be an influential factor on the prevalence of TB (*RR*, 1.00324). Specifically, with one unit increase of temperature, the risk of new TB case increased by 1.00324 times. This finding is similar to what Khaliq *et al*. [40] reported in a study on temporal and seasonal TB incidence patterns in Lahore, Pakistan from 2006 to 2013. They pointed out that temperature was significantly associated with TB incidence at the 0.01 level with *p* = 0.006 and *r* = 0.477. Mabaera *et al*. [41] also reported a similar relationship between temperature and TB in a study across four countries. The mechanism might be that higher temperatures help promote the activity of bacteria and improve their viability [42], which could also explain the phenomenon whereby a TB morbidity peak emerges in summer all over the world [43].

In addition to temperature, TB might also benefit from increased precipitation in that rainfall increases the living area of bacteria [19]. Desalu [44] reported in a retrospective analysis that pulmonary tuberculosis cases were higher in the rainy (wet) season than dry season. In our study, rainfall was also found to be statistically significant (*RR*, 1.01010); with one unit rise of the rainfall, the risk of new case of TB increases by 1.01010 unit.

Air pressure was also found to have a positive relationship with the prevalence of TB. With one unit rise of air pressure, the risk of new case of TB increases by 1.01007 unit. The reason might be that high air pressure leads to increased atmosphere flow, thus helping to spread airborne infections such as TB.

Wind speed was found to have a negative effect on TB prevalence. With one unit increase of wind speed, the risk of new TB cases decreases by 0.83518 unit. Theoretically, high wind speed could accelerate ventilation and thus dilute the concentration of bacteria, help reduce the risk of getting infected. Knibbs [45] performed air-exchange measurements to test whether ventilation can reduce risk of airborne disease (including tuberculosis and influenza) transmission, results showed that ventilation limited infection risks to 0.1%–3.6%.

No relationship between humidity and TB prevalence was found in our study. Although, humidity tended to be adjusted by rainfall and it may have an indirect influence on TB prevalence. However, this effect was considerably smaller than that of rainfall and was not statistically significant.

## 5. Limitations and Conclusions

In this study, data of tuberculosis on the province level was used to do analysis. In the future research, we want to collect data on the city level, which will provide a larger matrix of area connection (more detail spatial information). Besides, only data from year 2009 to 2013 was collected, so the time period could be extended in the future study.

## Figures and Tables

**Table 1:** Statistical models constructed based on Bayesian methodology.

Model	Estimated Relative Risk
UH	$\theta_{ij}=exp\left(\alpha_{0}+\alpha_{1j}+v_{i}\right)$
UH + autoregressive time effect	$\theta_{ij}=exp\left(\alpha_{0}+\alpha_{1j}+v_{i}+g_{i}\right)$
UH + time trend effect	$\theta_{ij}=exp\left(\alpha_{0}+v_{i}+\alpha_{1}time_{1j}\right)$
CH	$\theta_{ij}=exp\left(\alpha_{0}+\alpha_{1}time_{1j}+u_{i}+d_{i}time_{1j}\right)$
CH + UH	$\theta_{ij}=exp\left(\alpha_{0}+\alpha_{1}time_{1j}+u_{i}+v_{i}+d_{i}time_{1j}\right)$
CH + UH + temporal − spatial interaction	$\theta_{ij}=exp\left(\alpha_{0}+\alpha_{1}time_{1j}+u_{i}+v_{i}+psi_{ij}\right)$

Abbreviations: UH, uncorrelated effect model, different regions have no association with each other; CH, correlated effect model, one region has an effect on its adjacency; *α_1_*, intercept of each year; *v_i_*, uncorrelated effect; *u_i_*, correlated effect; *g_i_,* autoregressive time effect; *time_1j_*, time trend effect; *psi_ij_*, spatial-temporal interaction effect; *d_i_*, matrix of spatial connection among different provinces.

**Table 2:** Influential climate factors of prevalence for TB.

Variable	MEAN	SD	MC Error	*θ* (*RR*)	95% CI
a0	−0.29720	0.22050	0.00751	0.74289	0.40865–0.97988
a1	0.16160	0.10180	0.00347	1.17538	0.99146–1.46507
average temperature	0.00324	0.00108	5.6890E-7	1.00324	1.00150–1.00550
average rainfall	0.01005	1.669E-5	8.2300E-7	1.01010	1.01007–1.01013
average wind speed	−0.18010	0.00685	3.8360E-4	0.83518	0.93732–0.96138
average humidity	−0.02535	0.01565	8.7610E-5	0.97496	0.97181–1.01386
average air pressure	0.01002	2.4150E-5	8.2430E-5	1.01007	1.01003–1.01011
